# Diabetes mellitus and platelet reactivity in patients under prasugrel or ticagrelor treatment: an observational study

**DOI:** 10.1186/s12933-015-0232-1

**Published:** 2015-05-30

**Authors:** Dimitrios Alexopoulos, Chrysoula Vogiatzi, Katerina Stavrou, Niki Vlassopoulou, Angelos Perperis, Ioanna Pentara, Ioanna Xanthopoulou

**Affiliations:** Department of Cardiology, Patras University Hospital, University of Patras, Rion, Patras, Greece

**Keywords:** Antiplatelet agents, Antiplatelet drugs, Diabetes mellitus, Insulin, P2Y_12_ purinergic receptors

## Abstract

**Background:**

The influence of diabetes mellitus (DM) on platelet reactivity (PR) in prasugrel or ticagrelor treated patients is not well studied.

**Methods:**

In an observational study involving 777 patients with acute coronary syndrome undergoing percutaneous coronary intervention treated by either prasugrel 10 mg od (*n* = 315) or ticagrelor 90 mg bid (*n* = 462), platelet function was assessed using the VerifyNow P2Y_12_ function assay (in PRU) at one month post intervention.

**Results:**

In the overall population, ticagrelor and insulin-treated DM affected PR, with a decrease in log by 0.88 (corresponding to a 58 % decrease in PR) compared to prasugrel-treated patients (*p* < 0.001), and an increase in log by 0.26 (corresponding to a 30 % increase in PR) compared to non-diabetic patients (*p* = 0.01), respectively. PR in prasugrel-treated patients differed significantly by DM status: 70.0 (36.3-113.0) in non-diabetic vs 69.0 (44.5-115.3) in non insulin-treated diabetic vs 122.0 (69.0-161.0) in insulin-treated diabetic patients, p for trend = 0.01. No differences were observed in ticagrelor-treated patients. By multivariate analysis, in prasugrel-treated patients insulin-treated DM was the only factor predicting PR, with log of PR increased by 0.42 (corresponding to a 52 % increase in PR) compared to non-diabetic patients (*p* = 0.001). No factor was found to affect PR in ticagrelor-treated patients.

**Conclusions:**

Patients with insulin-treated DM treated with prasugrel post PCI have higher PR, than patients without DM or non insulin-treated diabetic patients treated with this drug. Ticagrelor treated patients have overall lower PR than patients on prasugrel, independent of DM status or insulin treatment.

**Trial registration:**

Clinical Trials Gov. NCT01774955

## Background

Patients with type 2 diabetes mellitus (DM) present with a prothrombotic state, for which platelet dysfunction is heavily implicated [1]. Despite a considerable variation of its antiplatelet effects among individuals, clopidogrel is the most commonly used P2Y_12_ receptor blocker in patients undergoing percutaneous coronary intervention (PCI), including those with DM [2]. High platelet reactivity (HPR) while on clopidogrel is more prevalent in diabetic compared with non- diabetic patients, with platelet reactivity (PR) levels and HPR frequency being highest among those patients requiring insulin therapy [3–5]. Moreover, DM presence has been identified as a strong predictor of major adverse cardiovascular events following PCI with stent implantation, while insulin treatment is recognized as an additional risk factor for stent thrombosis [6–9].

Prasugrel and ticagrelor are novel P2Y_12_ receptor blockers with more intensive and consistent than clopidogrel antiplatelet activity, introduced into clinical practice following the Trial to Assess Improvement in Therapeutic Outcomes by Optimizing Platelet Inhibition with Prasugrel–Thrombolysis in Myocardial Infarction (TRITON-TIMI) 38 and Platelet Inhibition and patient Outcomes (PLATO) trial, respectively [10, 11]. Novel agents exhibited better efficacy than clopidogrel, at cost of a higher bleeding potential. In the diabetic subpopulations, prasugrel and ticagrelor reduced the primary endpoint – a composite of cardiovascular death, myocardial infarction, or stroke - compared to clopidogrel by 30 % and 12 % respectively, without significant DM status-by-treatment interactions (p for interaction 0.09 and 0.49, respectively) [12, 13]. In TRITON-TIMI 38, a benefit of prasugrel over clopidogrel was observed regardless of whether subjects with DM were treated with insulin or not, although the absolute benefit was greater in insulin–treated DM patients (relative risk reduction by 37 % vs 26 %). Similarly, in PLATO trial ticagrelor, when compared with clopidogrel, reduced ischaemic events irrespective of diabetic status (*p* for interaction = 0.3), though the relative risk reduction was 22 % in insulin-treated vs 7 % in non insulin-treated patients.

In 2 previous randomized, pharmacodynamic studies, exclusively in diabetic patients, ticagrelor was found to provide lower PR compared to prasugrel [14, 15]. However, it is not clear whether DM is included among factors potentially influencing PR while on treatment with prasugrel or ticagrelor. DM was not reported among factors affecting PR under prasugrel in some, though not in all studies, while prasugrel pharmacokinetics was not influenced by DM status in TRITON-TIMI 38 [16–20]. Moreover, in patients under ticagrelor therapy, DM was not among factors influencing PR [19, 21]. Furthermore, the impact of insulin therapy on PR in DM patients treated with novel P2Y_12_ receptors blockers has not been previously analyzed. In the present study we aimed to analyze factors affecting PR in patients post PCI and under chronic maintenance dose of either prasugrel or ticagrelor, with particular emphasis on DM effect and the impact of insulin therapy.

## Methods

This is a cross-sectional, observational study in consecutive patients with acute coronary syndrome undergoing PCI who were discharged either on prasugrel 10 mg od or ticagrelor 90 mg bid and had platelet function assessment at one month post intervention. All patients participated in an ongoing study of platelet function testing for prediction of bleeding events (Clinical Trials Gov. NCT01774955), while part of PR data have been previously reported [19]. Platelet function testing was performed using the VerifyNow (Accumetrics Inc., San Diego, CA, USA) P2Y_12_ function assay, measured in P2Y12 reaction units (PRU). An intra-assay variability of 2.1 ± 1.3 % with a 6 % coefficient of variation has been described [22]. HPR was defined as >208 PRU [23]. Blood samples were obtained 2–4 h after the last drug dose. All patients were encouraged to receive prasugrel or first ticagrelor dose between 8 and 9 a.m. and second ticagrelor dose after 12 h. All patients were self-reported as compliant to therapy at one-month follow-up and received the same treatment as at discharge. Previously used definitions for DM, hypertension, dyslipidemia and myocardial infarction were employed [24–27].

### Statistical analysis

Categorical data are presented as frequencies and group percentages. Continuous data with normal and skewed distribution are presented as means ± standard deviation (SD) and medians (first to third quartile) respectively. One-way analysis of variance and Fisher’s exact test were used for comparison of normally distributed continuous and categorical data respectively. The Kruskal-Wallis test was used for comparison of skewed continuous data. Platelet reactivity differences between groups in the overall population and separately among ticagrelor and prasugrel-treated patients were analyzed via a generalized linear model with gamma distribution and logarithmic transformation of the dependent variable, DM status/type of treatment (insulin treated DM vs non-DM and non-insulin treated DM vs non-DM), male gender, statin use, proton pump inhibitor use, current smoking, hypertension, admission with ST-segment elevation myocardial infarction, creatinine clearance < 60 ml/min and treatment with ticagrelor (only for the overall population) as fixed effects and age and body mass index as covariates. All independent variables were simultaneously included in the model. The exponentiated coefficient represents the factor by which PR is multiplied.

All patients provided written informed consent. The study protocol conforms to the ethical guidelines of the 1975 Declaration of Helsinki as reflected in a priori approval by the institution’s human research committee.

## Results

Among 777 analyzed patients, 315 and 462 were on prasugrel and ticagrelor maintenance dose respectively. Patients’ characteristics by DM status and type of treatment are presented in Table 1.

**Table 1:** Demographic and clinical characteristics of patients by diabetic status and type of treatment

	No diabetes mellitus	Diabetes mellitus non insulin-treated	Diabetes mellitus insulin-treated	p-value
	N = 603	N = 132	N = 42	
Male gender	519 (86.1)	116 (87.9)	38 (90.5)	0.7
Age (years)	58.9 ± 11.2	63.4 ± 10.4	62.8 ± 10.5	<0.001
Body mass index (Kg/m^2^)	28.1 ± 4.2	29.2 ± 4.5	29.2 ± 5.7	0.01
Dyslipidemia	300 (49.8)	72 (54.5)	19 (45.2)	0.5
Hypertension	286 (47.4)	94 (71.2)	21 (50.0)	<0.001
Current smoking	39 (6.5)	4 (3.0)	2 (4.8)	0.3
Prior myocardial infarction	45 (7.5)	10 (7.6)	4 (9.5)	0.8
Prior CABG	9 (1.5)	2 (1.5)	2 (4.8)	0.3
Prior PCI	58 (9.6)	12 (9.1)	4 (9.5)	1.0
Prior stroke/TIA	10 (1.7)	6 (4.5)	1 (2.4)	0.2
Admission for PCI				0.2
STEMI	342 (56.7)	63 (47.7)	26 (61.9)	
NSTEMI	161 (26.7)	40 (30.3)	7 (16.7)	
Unstable angina	100 (16.6)	29 (22.0)	9 (21.4)	
Creatinine clearance <60 ml/min	51 (8.5)	18 (13.6)	11 (26.2)	0.001
P2Y_12_ receptor blocker at discharge and follow-up				0.7
Ticagrelor	363 (60.2)	76 (57.6)	23 (54.8)	
Prasugrel	240 (39.8)	56 (42.4)	19 (45.2)	
Other discharge medication*				
Aspirin 100 mg	598 (99.2)	131 (99.2)	42 (100)	1.0
Statin	595 (98.7)	128 (97.0)	40 (95.2)	0.09
Proton pump inhibitor	572 (94.9)	120 (90.9)	34 (81.0)	0.002
Beta-blocker	565 (93.7)	121 (91.7)	40 (95.2)	0.7
Nitrate	66 (10.9)	20 (15.2)	8 (19.0)	0.1

Data are expressed as means ± SD, medians (first to third quartiles) or n (%). CABG = coronary artery bypass grafting; NSTEMI = non ST segment elevation myocardial infarction; PCI = percutaneous coronary intervention; STEMI = ST segment elevation myocardial infarction; TIA = transient ischemic attack

*No patient was on oral anticoagulant treatment

In the overall population, 2 factors were found independently affecting PR at one month: i) Treatment with ticagrelor, with log of PR decreased by 0.88 (corresponding to a 58 % decrease in PR) compared to prasugrel-treated patients and ii) insulin-treated DM, with log of PR increased by 0.26 (corresponding to a 30 % increase in PR) compared to non-diabetic patients (Table 2).

**Table 2:** Multivariate analysis of platelet reactivity in the overall population

	Coefficient (SE)	t	95 % CI	Exponentiated coefficient*	p-value
Male gender	0.04 (0.09)	0.24	−0.13 to 0.21	1.04	0.6
Current smoking	−0.15 (0.12)	1.48	−0.39 to 0.09	0.86	0.2
-Insulin-treated diabetes mellitus (vs non-diabetic status)	0.26 (0.11)	6.03	0.053 to 0.47	1.30	0.01
-Non insulin-treated diabetes mellitus (vs non-diabetic status)	0.04 (0.07)	0.34	−0.10 to 0.18	1.04	0.6
Ticagrelor (vs prasugrel)	−0.88 (0.06)	217.9	−0.99 to −0.76	0.42	<0.001
Age	0.001 (0.003)	0.08	−0.005 to 0.007	1.001	0.8
Body mass index	0.006 (0.007)	0.88	−0.007 to 0.019	1.006	0.4
Creatinine clearance < 60 ml/min	0.10 (0.09)	1.09	−0.09 to 0.29	1.1	0.3
Statin	0.24 (0.18)	1.79	−0.11 to 0.60	1.27	0.2
Proton pump inhibitor	0.11 (0.12)	0.75	−0.14 to 0.35	1.11	0.4
Hypertension	0.04 (0.06)	0.41	−0.08 to 0.16	1.04	0.5
STEMI at admission	−0.11 (0.06)	3.10	−0.23 to 0.01	0.90	0.08

SE = standard error, CI = confidence interval, STEMI = ST-segment elevation myocardial infarction. *Exponentiated coefficient = e^^Coefficient^ is the factor by which PR on the original scale is multiplied

**Fig. 1:**
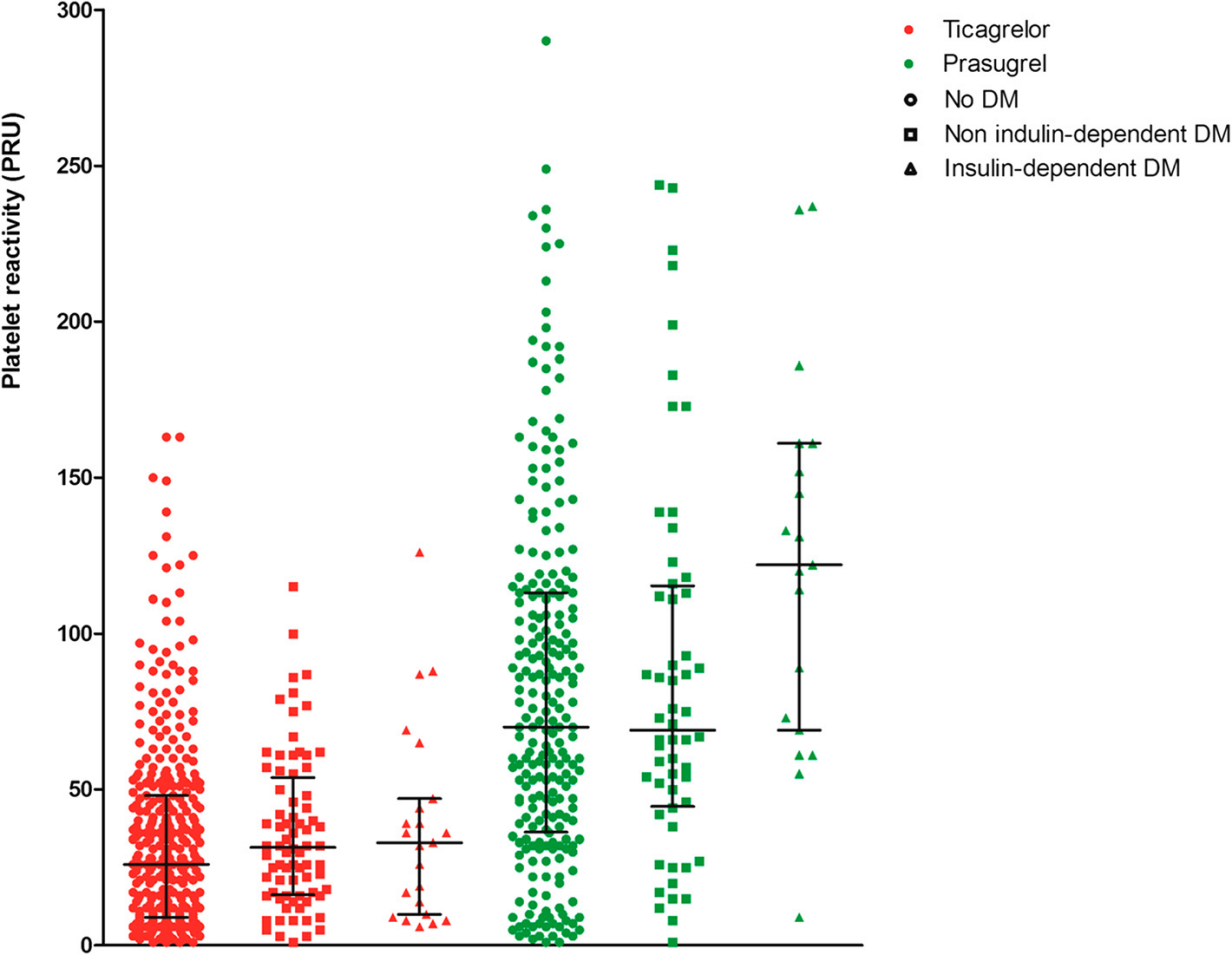
Patients’ individual platelet reactivity values by diabetic status and type of treatment separately for ticagrelor and prasugrel-treated patients; lines represent medians and error bars inter-quartile range. DM = diabetes mellitus

Patients’ individual PR values by DM status and type of treatment separately for ticagrelor and prasugrel-treated patients are presented in Fig. 1. Platelet reactivity (PRU) among prasugrel-treated patients differed significantly: 70.0 (36.3-113.0) in non diabetic vs 69.0 (44.5-115.3) in non insulin-treated diabetic vs 122.0 (69.0-161.0) in insulin-treated diabetic patients, p for trend = 0.01. In contrast, among ticagrelor-treated patients, PR did not differ significantly by DM status: 26.0 (9.0-48.0) in non-diabetic vs 31.5 (16.3-53.8) in non insulin-treated diabetic vs 33.0 (10.0-47.0) in insulin-treated diabetic patients, *p* = 0.1.

In the subgroup of prasugrel-treated patients, DM status had an overall significant effect on PR (*p* = 0.002). By multivariate analysis, insulin-treated DM was the only factor with a significant effect on PR, with log of PR increased by 0.42 (corresponding to a 52 % increase in PR) compared to non diabetic patients. Non-insulin treated DM had no such impact on PR (Table 3). No factor was found to affect PR in ticagrelor-treated patients (Table 4).

**Table 3:** Multivariate analysis of platelet reactivity in patients under prasugrel

	Coefficient (SE)	t	95 % CI	Exponentiated coefficient*	p-value
Male gender	0.07 (0.14)	0.25	−0.21 to 0.35	1.07	0.6
Current smoking	0.01 (0.16)	0.003	−0.31 to 0.33	1.01	0.9
-Insulin-treated diabetes mellitus (vs non-diabetic status)	0.42 (0.12)	12.0	0.18 to 0.65	1.52	0.001
-Non insulin-treated diabetes mellitus (vs non-diabetic status)	0.03 (0.11)	0.09	−0.19 to 0.25	1.03	0.8
Age	0.001 (0.004)	0.02	−0.007 to 0.008	1.001	0.9
Body mass index	0.02 (0.009)	3.35	−0.001 to 0.033	1.02	0.07
Creatinine clearance < 60 ml/min	−0.07 (0.18)	0.15	−0.42 to 0.28	0.93	0.7
Statin	−0.03 (0.38)	0.006	−0.77 to 0.71	0.97	0.9
Proton pump inhibitor	0.14 (0.19)	0.58	−0.22 to 0.50	1.15	0.4
Hypertension	0.07 (0.08)	0.88	−0.08 to 0.22	1.07	0.4
STEMI at admission	−0.12 (0.08)	2.0	−0.27 to 0.04	0.89	0.2

Abbreviations as in Table 2

**Table 4:** Multivariate analysis of platelet reactivity in patients under ticagrelor

	Coefficient (SE)	t	95 % CI	Exponentiated coefficient*	p-value
Male gender	0.04 (0.11)	0.15	−0.17 to 0.26	1.04	0.7
Current smoking	−0.19 (0.15)	1.54	−0.48 to 0.11	0.83	0.2
-Insulin-treated diabetes mellitus (vs non-diabetic status)	0.10 (0.18)	0.28	−0.26 to 0.45	1.11	0.6
-Non insulin-treated diabetes mellitus (vs non-diabetic status)	0.08 (0.10)	0.74	−0.11 to 0.27	1.08	0.4
Age	0.001 (0.005)	0.04	−0.008 to 0.01	1.001	0.9
Body mass index	−0.002 (0.01)	0.03	−0.02 to 0.02	0.998	0.9
Creatinine clearance < 60 ml/min	0.20 (0.12)	2.98	−0.03 to 0.43	1.22	0.08
Statin	0.33 (0.19)	2.99	−0.04 to 0.71	1.39	0.08
Proton pump inhibitor	0.07 (0.17)	0.18	−0.26 to 0.41	1.07	0.7
Hypertension	0.006 (0.09)	0.005	−0.17 to 0.18	1.006	0.9
STEMI at admission	−0.12 (0.09)	1.80	−0.29 to 0.05	0.89	0.2

Abbreviations as in Table 2

Among prasugrel-treated patients, HPR rates were 3.3 % (8/240) in non-diabetic, 7.1 % (4/56) in non insulin-treated diabetic and 10.5 % (2/19) in insulin-treated diabetic patients (*p* for trend = 0.1). No ticagrelor-treated patient presented with HPR.

## Discussion

In patients with acute coronary syndrome undergoing PCI and receiving maintenance prasugrel or ticagrelor therapy for 1 month, apart from a lower degree of PR provided by ticagrelor vs prasugrel, this study demonstrates that i) among prasugrel-treated patients, PR levels are clearly differentiated (higher) in insulin-treated diabetic patients, while they are similar between non-diabetic and non insulin-treated diabetic patients and ii) ticagrelor provides an homogeneous, very strong platelet inhibition, not influenced by DM status or insulin/non-insulin treatment.

Several recent studies have emphasized the complex interaction between DM and platelet function. A higher mean platelet volume was found in patients with prediabetes than in normal subjects, which is positively associated with fasting plasma levels [28]. In this cohort also, a common platelet antigen polymorphism [PLA1A2] of the gene encoding Glycoprotein IIIa has been associated with mortality when HbA1c is ranging from 5.5 % to 6.5 %, and maintenance of euglycemia and antiplatelet therapy are regarded as effective primary prevention measures [29]. Of note, in stable patients undergoing PCI, the variability of on-treatment platelet function and associated outcome is mainly influenced by clinical risk variables, including DM [30]. In addition, in type 2 diabetic patients, younger age is the most important predictor of high on-aspirin platelet reactivity [31]. Moreover, different anti-diabetic combination therapies seem to differentially affect platelet function. In metformin-treated type 2 diabetic patients, add-on therapy with pioglitazone was found to be more effective than glipizide for inhibiting platelet activation [32].

In line with previous reports, in the present study in a large cohort of patients under prasugrel or ticagrelor maintenance dose, including 174 patients with DM, and after adjusting for several factors, treatment with ticagrelor independently predicted lower PR [19, 33]. Of note, in a recent network meta-analysis ticagrelor was reported to achieve significantly lower on-treatment PR compared with prasugrel, with both being superior to clopidogrel standard or high dose [34]. Most importantly and, to our knowledge for the first time, the current analysis demonstrates a positive relation between insulin-treated DM and PR in patients receiving maintenance therapy with novel antiplatelet agents. In addition, this impact seems to be defined in the prasugrel-treated cohort.

### PR under prasugrel treatment

Previous studies of factors affecting PR while on prasugrel therapy provided conflicting or unclear results concerning the impact of DM on it, without analyzing the type of DM treatment effect [16–19]. Among 444 prasugrel-treated patients with acute coronary syndrome undergoing PCI and assessed 2 to 4 weeks after hospital discharge, patients with DM had higher vasodilator-stimulated phosphoprotein (VASP) index than non-DM patients, but this effect was not present in multivariate analysis [16]. In a previous analysis, by our group, of 234 patients under prasugrel maintenance dose, constituting part of the present cohort, and assessed by the VerifyNow, DM had a significant effect on PR with 36.3 % increase compared to non-DM patients [19]. In the present larger cohort, DM effect on prasugrel pharmacodynamics is further elucidated and seems to be mostly confined in insulin-treated diabetic patients.

Several explanations could be discussed for the above findings. Platelets of diabetic patients present with a decreased sensitivity to insulin, upregulation of the P2Y12 pathway and increased reactivity. Mechanisms like increased exposure to ADP, increased cytosolic levels of calcium, and increased platelet turnover may also be implicated in the response to P2Y_12_ receptor blockers in DM patients [1, 35]. These abnormalities are likely more pronounced in the insulin-treated diabetic patient [5] and may partially explain the observed impact on PR under prasugrel. Moreover, and concerning the other thienopyridine, clopidogrel, active metabolite kinetic profile-and to a lesser degree platelet dysfunction-seem to be mostly responsible for the overall impaired platelet P2Y_12_ receptor blockade mediated in DM patients [4]. However, in a pharmacokinetic analysis in 1159 patients participating in the TRITON-TIMI 38 trial, the systemic exposure to prasugrel was not appreciably affected by DM status [20]. Of note, a separate analysis of prasugrel active metabolite kinetics for insulin-treated diabetic was not performed. It seems therefore that our findings of a neutral and a negative impact of non insulin-treated DM and insulin-treated DM on PR respectively, could be attributed to platelet dysfunction in the latest high risk subgroup which cannot be entirely overcome by the potent antiplatelet thienopyridine prasugrel. In the largest so far pharmacodynamic study of patients under prasugrel maintenance treatment, an HPR rate of 10-15 % has been described, although not stratified by DM status [36]. In the present study, HPR rate under prasugrel therapy was slightly lower, while a trend for a progressive increase according to DM status and type of treatment was apparent.

### PR under ticagrelor treatment

In a patient-level data meta-analysis of 8 studies involving 445 ticagrelor-treated patients, DM did not emerge as a factor predicting PR, although independently associated with lower probability for PR <10 PRU [21]. In line, in the present analysis, among ticagrelor-treated patients, no sign of any influence on PR by DM was seen, even in the high risk group of insulin-treated diabetic patients. Hence, insulin-treated DM status may impact the thienopyridines clopidogrel and prasugrel action, but not ticagrelor’s one, which is a cyclopentyltriazolo-pyrimidine. Moreover, ticagrelor is a reversible P2Y_12_ ADP receptor blocker, administered twice daily, which may be more optimal for providing consistent inhibition for patients with high platelet turnover rates such as those with DM [37, 38]. Although it is unknown whether DM status modulates plasma levels of ticagrelor and its metabolite (AR- C124910XX), the described absence of any impact on its pharmacodynamics makes it extremely unlikely. In no case we advocate absence of platelet abnormalities following ticagrelor treatment. Only 1 signaling pathway, the P2Y12 one, is blocked by ticagrelor, leaving multiple other signaling pathways, many known to be upregulated in DM patients, uninhibited [1].

### Clinical relevance

As the great majority of prasugrel- and all of ticagrelor-treated patients respectively had PR levels below the threshold known to be accompanied by ischemic events, our results do not provide a potential explanation why diabetic patients and particularly insulin-treated ones have worse outcomes, despite treatment with novel antiplatelet agents. The observed detrimental impact of insulin-treated DM on PR under prasugrel may simply reflect platelet dysfunction of unclear clinical significance, considering the excellent performance of this agent in insulin-treated DM in TRITON-TIMI 38- with a 37 % reduction in the primary endpoint compared to clopidogel [10]. Furthermore, the described impact of insulin-treated DM on PR under prasugrel is not in discordance with the relative greater benefit provided by prasugrel in the insulin-treated diabetic cohort versus non-insulin treated diabetic [12]. This, most likely reflects a considerable ‘weakness’ of clopidogrel in insulin-treated diabetic patients [5]. In the DM subgroup of the TRITON-TIMI-38 trial, there was no difference in major bleeding between prasugrel and clopidogrel treated patients, regardless DM treatment type [12]. Even with the insulin detrimental effect, PR values in our prasugrel-treated DM patients were much lower than provided by clopidogrel [36] and are, therefore, unlikely to provide an explanation for bleeding rates observed in DM cohort of TRITON-TIMI-38 trial.

HPR under prasugrel, even in insulin-treated diabetic patients was very low. Nevertheless, its identification might enable a better understanding of their individual risk profile and allow the future development of targeted treatment strategies for these patients. Overall, our study offers a better understanding of DM status and treatment influence on novel antiplatelets’ pharmacodynamic behavior, while it demonstrates a differential effect of insulin-treated DM on PR according to the administered antiplatelet agent.

### Limitations

This is a cross-sectional study of independent groups and suffers from the obvious limitations of a nonrandomized trial. In an attempt to account for these limitations, we made adjustment for several clinical variables potentially affecting PR although additional bias cannot be entirely excluded. Although HbA1C levels were not measured, their association with PR has been seriously disputed [5, 39]. Genetic variants of the insulin receptor substrate associated with a hyper-reactive platelet phenotype were not analyzed [40]. A larger number of patients would increase study’s power, resulting in more precise estimates. Although the dynamic range of the VerifyNow assay appears to be narrower than that of light transmittance aggregometry, measurements of platelet inhibition while on prasugrel or ticagrelor using the 2 methods are well correlated [41, 42].

## Conclusions

Among acute coronary syndrome patients undergoing PCI and receiving maintenance therapy with prasugrel, insulin-treated diabetic patients have higher PR than patients without DM or non insulin-treated diabetic patients. Ticagrelor treated patients have overall lower PR than patients on prasugrel, independent of DM status or insulin treatment. Further study for treatment individualization according to these findings is guaranteed.

## References

[1] Ferreiro JL, Angiolillo DJ (2011). Diabetes and antiplatelet therapy in acute coronary syndrome. Circulation.

[2] Angiolillo DJ, Fernandez-Ortiz A, Bernardo E, Ramírez C, Sabaté M, Jimenez-Quevedo P (2005). Platelet function profiles in patients with type 2 diabetes and coronary artery disease on combined aspirin and clopidogrel treatment. Diabetes.

[3] Geisler T, Anders N, Paterok M, Langer H, Stellos K, Lindemann S (2007). Platelet response to clopidogrel is attenuated in diabetic patients undergoing coronary stent implantation. Diabetes Care.

[4] Angiolillo DJ, Jakubowski JA, Ferreiro JL, Tello-Montoliu A, Rollini F, Franchi F (2014). Impaired responsiveness to the platelet P2Y12 receptor antagonist clopidogrel in patients with type 2 diabetes and coronary artery disease. J Am Coll Cardiol.

[5] Angiolillo DJ, Bernardo E, Ramirez C, Costa MA, Sabaté M, Jimenez-Quevedo P (2006). Insulin therapy is associated with platelet dysfunction in patients with type 2 diabetes mellitus on dual oral antiplatelet treatment. J Am Coll Cardiol.

[6] Angiolillo DJ, Bernardo E, Sabate M, Jimenez-Quevedo P, Costa MA, Palazuelos J (2007). Impact of platelet reactivity on cardiovascular outcomes in patients with type 2 diabetes mellitus and coronary artery disease. J Am Coll Cardiol.

[7] Machecourt J, Danchin N, Lablanche JM, Fauvel JM, Bonnet JL, Marliere S (2007). Risk factors for stent thrombosis after implantation of sirolimus-eluting stents in diabetic and nondiabetic patients: the EVASTENT Matched-Cohort Registry. J Am Coll Cardiol.

[8] Taniwaki M, Stefanini GG, Silber S, Richardt G, Vranckx P, Serruys PW (2014). 4-year clinical outcomes and predictors of repeat revascularization in patients treated with new-generation drug-eluting stents: a report from the RESOLUTE All-Comers trial (A Randomized Comparison of a Zotarolimus-Eluting Stent With an Everolimus-Eluting Stent for Percutaneous Coronary Intervention). J Am Coll Cardiol.

[9] Palmerini T, Dangas G, Mehran R, Caixeta A, Généreux P, Fahy MP (2011). Predictors and implications of stent thrombosis in non-ST-segment elevation acute coronary syndromes: the ACUITY Trial. Circ Cardiovasc Interv.

[10] Wiviott SD, Braunwald E, McCabe CH, Montalescot G, Ruzyllo W, Gottlieb S (2007). Prasugrel versus clopidogrel in patients with acute coronary syndromes. N Engl J Med.

[11] Wallentin L, Becker RC, Budaj A, Cannon CP, Emanuelsson H, Held C (2009). Ticagrelor versus clopidogrel in patients with acute coronary syndromes. N Engl J Med.

[12] Wiviott SD, Braunwald E, Angiolillo DJ, Meisel S, Dalby AJ, Verheugt FW (2008). Greater clinical benefit of more intensive oral antiplatelet therapy with prasugrel in patients with diabetes mellitus in the trial to assess improvement in therapeutic outcomes by optimizing platelet inhibition with prasugrel-Thrombolysis in Myocardial Infarction 38. Circulation.

[13] James S, Angiolillo DJ, Cornel JH, Erlinge D, Husted S, Kontny F (2010). Ticagrelor vs. clopidogrel in patients with acute coronary syndromes and diabetes: a substudy from the PLATelet inhibition and patient Outcomes (PLATO) trial. Eur Heart J.

[14] Alexopoulos D, Xanthopoulou I, Mavronasiou E, Stavrou K, Siapika A, Tsoni E (2013). Randomized assessment of ticagrelor versus prasugrel antiplatelet effects in patients with diabetes. Diabetes Care.

[15] Laine M, Frère C, Toesca R, Berbis J, Barnay P, Pansieri M (2014). Ticagrelor versus prasugrel in diabetic patients with an acute coronary syndrome. A pharmacodynamic randomised study. Thromb Haemost.

[16] Cayla G, Cuisset T, Silvain J, O’Connor SA, Kerneis M, Castelli C (2013). Prasugrel monitoring and bleeding in real world patients. Am J Cardiol.

[17] Mayer K, Orban M, Bernlochner I, Braun S, Schulz S, Gross L (2015). Predictors of antiplatelet response to prasugrel during maintenance treatment. Platelets.

[18] Alexopoulos D, Xanthopoulou I, Perperis A, Siapika A, Stavrou K, Tsoni E (2013). Factors affecting residual platelet aggregation in prasugrel treated patients. Curr Pharm Des.

[19] Alexopoulos D, Stavrou K, Koniari I, Gkizas V, Perperis A, Kontoprias K (2014). Ticagrelor vs prasugrel one-month maintenance therapy: impact on platelet reactivity and bleeding events. Thromb Haemost.

[20] Wrishko RE, Ernest CS, Small DS, Ni L, Winters KJ, Farid NA (2009). Population pharmacokinetic analyses to evaluate the influence of intrinsic and extrinsic factors on exposure of prasugrel active metabolite in TRITON-TIMI 38. J Clin Pharmacol.

[21] Alexopoulos D, Xanthopoulou I, Storey RF, Bliden KP, Tantry US, Angiolillo DJ (2014). Platelet reactivity during ticagrelor maintenance therapy: a patient-level data meta-analysis. Am Heart J.

[22] Patti G, Nusca A, Mangiacapra F, Gatto L, D’Ambrosio A, Di Sciascio G (2008). Point-of-care measurement of clopidogrel responsiveness predicts clinical outcome in patients undergoing percutaneous coronary intervention results of the ARMYDA-PRO (Antiplatelet therapy for Reduction of MYocardial Damage during Angioplasty-Platelet Reactivity Predicts Outcome) study. J Am Coll Cardiol.

[23] Price MJ, Angiolillo DJ, Teirstein PS, Lillie E, Manoukian SV, Berger PB (2011). Platelet reactivity and cardiovascular outcomes after percutaneous coronary intervention: a time-dependent analysis of the Gauging Responsiveness with a VerifyNow P2Y12 assay: Impact on Thrombosis and Safety (GRAVITAS) trial. Circulation.

[24] Reiner Z, Catapano AL, De Backer G, Graham I, Taskinen MR, Wiklund O (2011). ESC/EAS Guidelines for the management of dyslipidaemias: the Task Force for the management of dyslipidaemias of the European Society of Cardiology (ESC) and the EuropeanAtherosclerosis Society (EAS). Eur Heart J.

[25] Thygesen K, Alpert JS, Jaffe AS, Simoons ML, Chaitman BR, White HD (2012). Third universal definition of myocardial infarction. Eur Heart J.

[26] Mancia G, Fagard R, Narkiewicz K, Redon J, Zanchetti A, Böhm M (2013). 2013 ESH/ESC guidelines for the management of arterial hypertension: the Task Force for the Management of Arterial Hypertension of the European Society of Hypertension (ESH) and of the European Society of Cardiology (ESC). Eur Heart J.

[27] Rydén L, Grant PJ, Anker SD, Berne C, Cosentino F, Danchin N (2013). ESC Guidelines on diabetes, pre-diabetes, and cardiovascular diseases developed in collaboration with the EASD: the Task Force on diabetes, pre-diabetes, and cardiovascular diseases of the European Society of Cardiology (ESC) and developed in collaboration with the European Association for the Study of Diabetes (EASD). Eur Heart J.

[28] Shimodaira M, Niwa T, Nakajima K, Kobayashi M, Hanyu N, Nakayama T (2013). Correlation between mean platelet volume and fasting plasma glucose levels in prediabetic and normoglycemic individuals. Cardiovasc Diabetol.

[29] Stratmann B, Xu T, Meisinger C, Menart B, Roden M, Herder C (2014). PLA1A2 platelet polymorphism predicts mortality in prediabetic subjects of the population based KORA S4-Cohort. Cardiovasc Diabetol.

[30] Droppa M, Tschernow D, Müller KA, Tavlaki E, Karathanos A, Stimpfle F (2015). Evaluation of clinical risk factors to predict high on-treatment platelet reactivity and outcome in patients with stable coronary artery disease (PREDICT-STABLE). PLoS One.

[31] Kaplon-Cieslicka A, Postula M, Rosiak M, Peller M, Kondracka A, Serafin A (2014). Younger age, higher body mass index and lower adiponectin concentration predict higher serum thromboxane B2 level in aspirin-treated patients with type 2 diabetes: an observational study. Cardiovasc Diabetol.

[32] Xiao CC, Ren A, Yang J, Ye SD, Xing XN, Li SM (2015). Effects of pioglitazone and glipizide on platelet function in patients with type 2 diabetes. Eur Rev Med Pharmacol Sci.

[33] Alexopoulos D, Galati A, Xanthopoulou I, Mavronasiou E, Kassimis G, Theodoropoulos KC (2012). Ticagrelor versus prasugrel in acute coronary syndrome patients with high on-clopidogrel platelet reactivity following percutaneous coronary intervention: a pharmacodynamic study. J Am Coll Cardiol.

[34] Lhermusier T, Lipinski MJ, Tantry US, Escarcega RO, Baker N, Bliden KP (2015). Meta-analysis of direct and indirect comparison of ticagrelor and prasugrel effects on platelet reactivity. Am J Cardiol.

[35] Ferreira IA, Mocking AI, Feijge MA, Gorter G, van Haeften TW, Heemskerk JW (2006). Platelet inhibition by insulin is absent in type 2 diabetes mellitus. Arterioscler Thromb Vasc Biol.

[36] Gurbel PA, Erlinge D, Ohman EM, Neely B, Neely M, Goodman SG (2012). Platelet function during extended prasugrel and clopidogrel therapy for patients with ACS treated without revascularization: the TRILOGY ACS platelet function substudy. JAMA.

[37] Husted S, van Giezen JJ (2009). Ticagrelor: the first reversibly binding oral P2Y12 receptor antagonist. Cardiovasc Ther.

[38] DiMinno G, Silver MJ, Cerbone AM, Murphy S (1986). Trial of repeated low-dose aspirin in diabetic angiopathy. Blood.

[39] Mangiacapra F, Peace AJ, Wijns W, Barbato E (2011). Lack of correlation between platelet reactivity and glycaemic control in type 2 diabetes mellitus patients treated with aspirin and clopidogrel. J Thromb Thrombolysis.

[40] Angiolillo DJ, Bernardo E, Zanoni M, Vivas D, Capranzano P, Malerba G (2011). Impact of insulin receptor substrate-1 genotypes on platelet reactivity and cardiovascular outcomes in patients with type 2 diabetes mellitus and coronary artery disease. J Am Coll Cardiol.

[41] Jakubowski JA, Payne CD, Li YG, Brandt JT, Small DS, Farid NA (2008). The use of the VerifyNow P2Y12 point-of-care device to monitor platelet function across a range of P2Y12 inhibition levels following prasugrel and clopidogrel administration. Thromb Haemost.

[42] Jeong YH, Bliden KP, Antonino MJ, Park KS, Tantry US, Gurbel PA (2012). Usefulness of the VerifyNow P2Y12 assay to evaluate the antiplatelet effects of ticagrelor and clopidogrel therapies. Am Heart J.

